# Phenotypic and Genotypic Characterization and Correlation Analysis of Pea (*Pisum sativum* L.) Diversity Panel

**DOI:** 10.3390/plants11101321

**Published:** 2022-05-16

**Authors:** Ana Uhlarik, Marina Ćeran, Dalibor Živanov, Radu Grumeza, Leif Skøt, Ellen Sizer-Coverdale, David Lloyd

**Affiliations:** 1 Institute of Field and Vegetable Crops, Maksima Gorkog 30, 21000 Novi Sad, Serbia; marina.ceran@ifvcns.ns.ac.rs (M.Ć.); dalibor.zivanov@ifvcns.ns.ac.rs (D.Ž.); 2 Agro Seed Research, Nijverheidslaan 1506, 3660 Oudsbergen, Belgium; r.grumeza@stormseeds.com; 3 Institute of Biological, Environmental & Rural Sciences (IBERS), Aberystwyth University, Penglais, Ceredigion, Aberystwyth SY23 3DA, UK; lfs@aber.ac.uk (L.S.); eys@aber.ac.uk (E.S.-C.); david.lloyd@germinal.com (D.L.)

**Keywords:** pea, seed yield, protein, yield components, correlation, heritability

## Abstract

Phenotypic and genotypic characterization were performed to assess heritability, variability, and seed yield stability of pea genotypes used in breeding to increase the pea production area. A European pea diversity panel, including genotypes from North America, Asia, and Australia consisting of varieties, breeding lines, pea, and landraces was examined in 2019 and 2020 in Serbia and Belgium using augmented block design. The highest heritability was for thousand seed weight; the highest coefficient of variation was for seed yield. The highest positive correlation was between number of seeds per plant and number of pods per plant; the highest negative correlation was between seed yield and protein content. Hierarchical clustering separated pea germplasm based on use and type. Different Principal component analysis grouping of landraces, breeding lines, and varieties, as well as forage types and garden and dry peas, confirms that there was an apparent decrease in similarity between the genotypes, which can be explained by their different purposes. Pea breeding should be focused on traits with consistent heritability and a positive effect on seed yield when selecting high-yielding genotypes, and on allowing for more widespread use of pea in various agricultural production systems.

## 1. Introduction

Pea (*Pisum sativum* L.) is considered to be the second most important protein crop after soybean, with a total production area of 2,436,344 ha in Europe in 2020 [1]. Despite the fact that the production area under peas has increased by 3.6% in the past five years [1], the demand for new sources of protein continues to grow [2], as total protein consumption in Europe, including plant-derived proteins, is about 70% higher than recommended [3]. To meet growing demand for quality protein while reducing reliance on imported plant protein from outside of Europe, improvement of the yield of protein crops produced in the region is desirable [4,5]. Protein content in pea as quoted in literature ranges from 16% to 30.9% [6,7]. In organic production systems in the European Union, peas are considered an important plant species because they are a source of biologically-fixed nitrogen and provide high-quality animal feed that is rich in crude protein and minerals [8]. Although soybean is one of the primary sources of plant-based protein, the advantages of growing peas over soybeans are its wider geographical area of cultivation and its ability to thrive in colder climates. Soybean, unlike pea, is one of the significant food allergens [9]; moreover, the fact that pea grain, unlike soybeans, can be used directly in the diet without prior heat treatment is essential, as this simplifies use and reduces processing costs. Combined sowing of peas with cereals is increasingly used in practice to improve the efficiency of land and water resources, nutrients, and solar radiation [8]. Furthermore, peas can also be grown as green manure [10] and as a forage crop, especially in regions where climatic conditions are not favorable for a good seed yield [11]. Their adaptability to a broad range of environmental conditions makes them an excellent cover crop due to the short vegetation season [12] and can improve productivity in successor crops. Therefore, there are great needs and benefits for increasing the production area under peas.

The fundamental goal of pea breeders is to increase seed yield to maximize plant productivity and allow for more widespread use of pea in various agricultural production systems. Seed yield is a complex trait that is quantitatively inherited, and its expression depends on genetic factors, environment, and interaction (GxE) [13]. Many studies have shown a significant influence of environment and genotype-by-environment (GxE) interaction on seed yield and the yield components of their phenotypic performance [14,15,16]. Genetic variability is a prerequisite in increasing the seed yield, since it helps peas adapt to changing environmental conditions and is the source of variation and raw material for yield improvement. In this type of study, seed availability can be a limiting factor, as variability testing requires a large number of genotypes (lines or populations). In many of those, there is generally little seed material. Furthermore, the logistics of fully replicated trials can be prohibitive in terms of space. This can be solved by using an augmented trial design [17], which requires a significantly smaller number of seeds, giving access to a broader choice for populations in germplasm collections than when using traditional experiment designs. Assessment of genetic variability is essential for efficient parent selection in breeding programs and long-term selection gain [18]. The broad cultivation range of pea and reliable pea yield stability [19] is associated with a broad genetic variation observed at the phenotypic and molecular levels [13]. Before initiating any effective selection program, it is necessary to know the association of various traits with yield and with each other, as a negative association between the desired attributes under selection may result in genetic slippage. Consequently, this could limit the genetic advance of the yield, which represents the end product of many correlated characters. An understanding of the influence of individual contributors on seed yield informs the breeder on how effective selection can take place. It is considered that yield selection can be more effective when it is based on component traits that are highly heritable and positively correlated [20].

The objectives of this study were to examine the phenotypic and genotypic variability in a set of diverse pea genotypes using morphological and agronomic traits, assess heritability, discover the relationships among seed yield components of pea genotypes, identify germplasm structure using correlation analysis, and determine sensitivity to environmental conditions and possible GxE interaction for yield and yield components. This research aimed to assess the phenotypic diversity of studied pea germplasm in order to enrich the breeding collection of the pea used in Western and Central Europe.

## 2. Results

### 2.1. Phenotypic Evaluation

Descriptive statistics and Pearson’s Correlation were conducted on morphological traits of analyzed pea genotypes. The studied features were number of grains per pod (GPP), number of pods per plant (PPP), number of seeds per plant (SPP), flowering duration (FD), plant height (PH), plot lodging (PL), pod length (PoL), thousand seed weight (TSW), seed weight per plant (SWPP), seed yield (SY), and protein content (PC).

According to the results of experiments at IFVCNS, Serbia, extensive phenotypic variation was observed in the analyzed pea panel. Means for PPP, SPP, SWPP, and PH were significantly lower in 2020 than in 2019, which is likely to be the consequence of drought in the flowering period in May 2020 (Table S1). In contrast, GPP, PoL, TSW, and SY were higher in 2020 than 2019. For all characteristics, statistically significant differences were observed for mean values between two seasons, except for PL and FD. The highest coefficients of variation (CV) were observed for SY and TSW in both years (42 and 36%, and 31 and 30%, respectively). High variation was also evident in SWPP and FD in both years (21–23%), while for PH and PL, higher values were recorded in the 2020 season (Table 1). The lowest CV was observed for PC (5%) in both years. The broad-sense heritability estimates were moderate for GPP, PPP, SPP, and SWPP and high for FD, PH, PoL, TSW, SY, and PC, which indicates that genetic constituents are the primary source of these traits. An exception was PL, whose heritability was significantly higher in 2020. The highest heritability of 96.6% and 97.7% were estimated for TSW in 2019 and 2020, respectively. Heritability was consistent between years for SPP, PH, PoL, TSW, SY, and PC and inconsistent for GPP, PPP, and PL (Table 1). Inconsistency between years for given traits could indicate their higher sensitivity to environmental conditions.

Results recorded at Agro Seed, Belgium, had mean SPP, PH and SY significantly lower in 2019 than in 2020. In contrast, TSW was higher in 2019 than in 2020, which is possibly explicable by higher temperatures observed during the pod filling period (Table S2). For all characteristics, statistically, significant differences were observed for mean values between two seasons for all traits, except for GPP. The highest coefficients of variation (CV) were observed for PL and SY in 2019 (37 and 31%) and for SY and GPP (31 and 28%) in 2020 (Table 2). High variation was also evident in PH and TSW in both years (16–31%). The coefficient of variability for all traits was generally higher in 2019. The lowest CV in 2019 was observed for PC (5%) and in 2020 for PC (6%).

The broad-sense heritability estimates were moderate to high for all traits, indicating that genetic constituents are the primary source of these traits. The highest heritability of 89.5% was estimated for TSW in 2019 and 88.5% for PC in 2020, respectively. Heritability was consistent between years for GPP, SPP, PoL and TSW, SY, and PC, and inconsistent for PPP, FL, PH, PL, and SWPP.

### 2.2. Correlation Analysis between Traits

The correlation coefficients for the IFVCNS trial between different traits are shown in Table 3. SWPP had positive and significant correlation with a great number of traits (GPP, PPP, SPP, PoL, TSW, and SY), but was highly negatively correlated with PC (−0.46); PC was also negatively correlated with SY and TSW. Furthermore, TSW was negatively correlated with GPP, PPP, and SPP, and positively with SY and PoL. Correlation between the remaining pairs of traits was mainly low and non-significant in both years.

The correlation coefficients for the Agro Seed trial are shown in Table 4. Trait SWPP had positive correlation with all the traits (insignificant for GPP, FD, and PC). Trait SPP had significant positive correlation with FD and PH. Traits SY and PC were positively correlated (0,14). PC was also highly positively correlated with SPP.

The differences between the two locations, regarding correlations between traits, were no significant correlation of GPP with FD, PH, PL, PoL, and SWPP in the Agro Seed trial, while the IFVCNS trial showed high correlations for these traits. Furthermore, in the Agro Seed trial there was no correlation between SY and traits FD and PL, while in the IFVCNS trial, there was no correlation between PC and SPP. The main difference between locations was in the correlation between PC and SY; IFVCNS trial showed negative correlation, while Agro Seed trial showed positive correlation.

### 2.3. Correlation of Traits between Two Years

**Figure 1:**
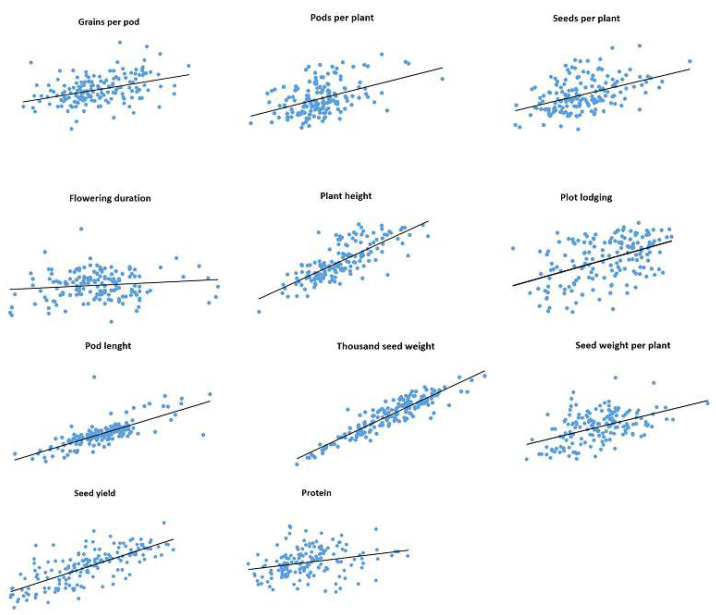
Correlations and linear regressions between data collected on the same pea genotypes (165) grown in two years (2019 and 2020) at IFVCNS, Serbia.

**Figure 2:**
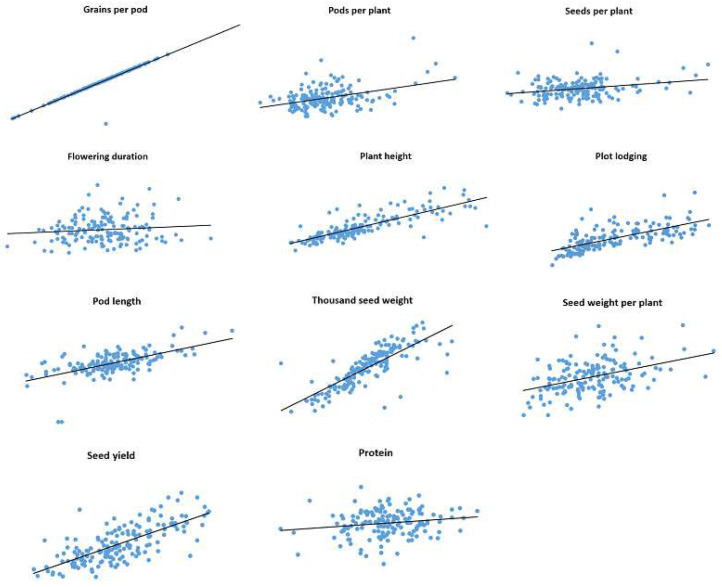
Correlations and linear regressions between data collected on the same pea genotypes (165) grown in two years (2019 and 2020) at Agro Seed, Belgium.

Correlations between trait values observed in 2019 and 2020 for IFVCNS and Agro Seed were graphically represented using scatter diagrams (Figure 1 and Figure 2). For all examined traits, linear regressions in pairwise comparisons between years revealed positive correlations (Table S3).

For the IFVCNS trial, correlations between seasons were mostly highly significant, except for flowering duration (FD). The strongest correlation was noticed for TSW (0.92), PH (0.77), and PoL (0.75), followed by GPP (0.60), SWPP (0.48), PL (0.46), SPP (0.45), and PPP (0.44), which had lower Pearson’s correlation coefficients (r). FD and PC had the lowest correlation between years (0.13 and 0.07, respectively).

For the Agro Seed trial, the strongest correlation was noticed for GPP (0.96), PH (0.67), TSW (0.64), and the lowest correlation was for SPP (0.07) due to the high virus presence in 2020 (data not shown), FD (0.01), and PC (0.03). PC had a low correlation between localities (0.27 at IFVCNS and 0.17 at Agro Seed).

### 2.4. Multivariate Analysis

**Figure 3:**
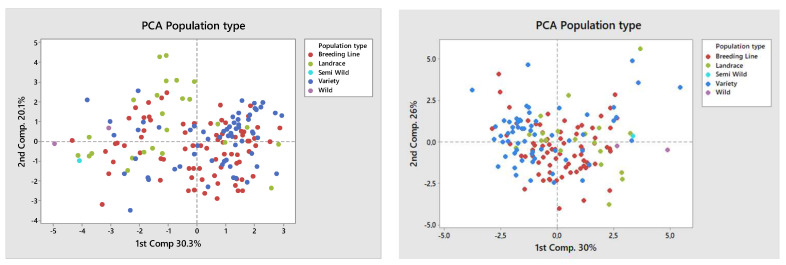
Principal component analysis (PCA) of the broad phenotypic diversity of the investigated pea panel composed by analyzed genotypes, classified by plant type. (**Left**): IFVCNS, Serbia site results; (**Right**): Agro Seed, Belgium site results.

**Figure 4:**
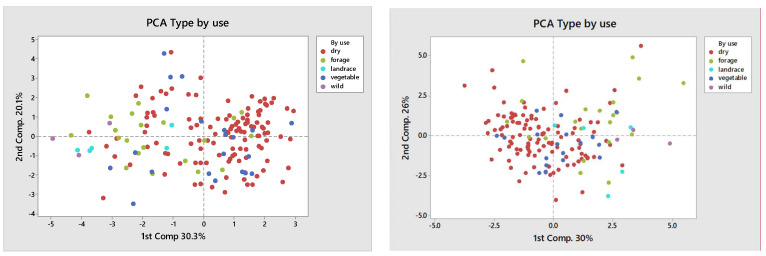
Principal component analysis (PCA) of the broad phenotypic diversity of the investigated pea panel composed by analyzed genotypes, classified by variety type by usage. (**Left**): IFVCNS, Serbia site results; (**Right**): Agro Seed, Belgium site results.

Multivariate analysis was performed based on all examined traits for both trial sites using mean values of both years to explore the population structure of the pea genotypes of different plant types (Figure 3), as well as of the variety type by use (Figure 4). The PCA gave a graphical representation of the broad phenotypic diversity of the investigated pea panel. Based on the results from IFVCNS, the first principal component accounted for 30.3% of the variance and showed no apparent clustering of the genotypes based on population type. The second principal component accounted for 20.1% of the variance and showed clustering of landrace types and overlapping of varieties and most breeding lines. Based on the results from Agro Seed, the first principal component accounted for 30% of the variance and showed some clustering of the breeding lines. In comparison, the second principal component accounted for 26% of the variance and showed a less clear subdivision of varieties. A large number of the breeding lines and varieties originated from North America and Serbia, while most of the landraces originated from Sweden (Table S4).

Morpho-phenological data in both trial sites did not allow completely clear subdivision between or within gene pools.

PCA analysis based on the variety type on both locations showed a higher prevalence of dry pea type and showed that accessions of dry pea type were generally clustered according to their use. However, it did not provide a clearer subdivision of pea genotypes by use.

Although PCA analysis did not show a clear grouping of genotypes based on plant type, an analysis of variance was conducted to determine possible differences within the germplasm. Analysis of variance for results at IFVCNS indicated a statistically significant difference among pea genotypes for the following traits: GPP, PH, PoL, and TSW in both years (Table 5). Furthermore, differences were observed for SPP and SWPP only in 2019. Analysis of variance for results at Agro Seed indicated a statistically significant difference among pea genotypes for the following traits: PH and TSW in both years, and PC, PoL, and PC in 2020.

### 2.5. Genetic Distance and Hierarchical Clustering

**Figure 5:**
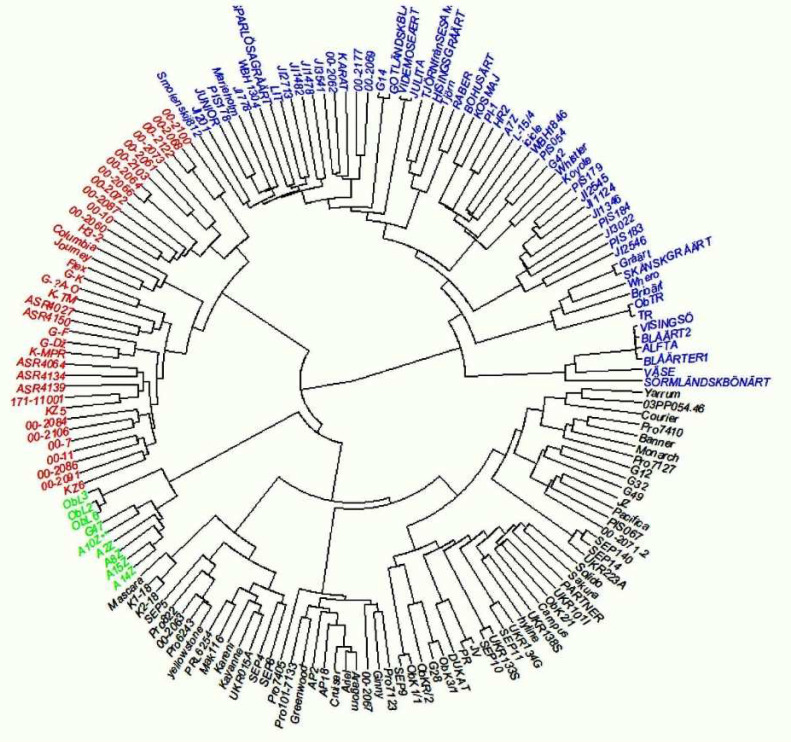
Hierarchical clustering of 156 pea accessions used in the experiment. The accessions were classified into four groups based on the clustering.

This work was part of a more extensive study on genetic diversity and phenotypic variation in yield and protein content of pea accessions in Europe. After filtering, markers with a Gentrain score (Illumina quality measure) below 0.7 and a minimum allele frequency of 0.05–a total of 11,693 polymorphic markers—were left in the panel of 165 accessions used here. The genetic distance matrix was based on all the 16,693 markers and the phenotypic distance matrices were based on all the phenotypic traits measured in this study. The hierarchical grouping chart showed four groups (Figure 5). The first group (green) consists of nine genotypes, exclusively of the breeding line population type and dry type of use, originating from Serbia. The second group (red) consists of 36 genotypes, mainly of the breeding line population type and dry type of use, with some exceptions of the vegetable type. In this group, vegetable pea lines, as expected, are clustered very closely together (mainly the 00 lines, Table S4); however, a set of 00 lines shows introgressions from other types of peas. A number of forage peas are also part of this genetic group (Flex, H3) The third group (blue) consists of 57 genotypes, mainly landraces, forage, wild/semi-wild population, and dry types of use. Clustering showed that some of the landraces are very closely related. The fourth group (black) consists of 63 genotypes, mostly varieties and dry types of use. This group consists of modern dry varieties, which are related and grouped together.

A Mantel test was performed to test the correlations between the genetic distance matrix and the phenotypic distance matrices at the two sites and years (Table 6). There were significant correlations at both sites and in both years. However, the correlations were highest for the two seasons at IFVCNS, Serbia.

**Figure 6:**
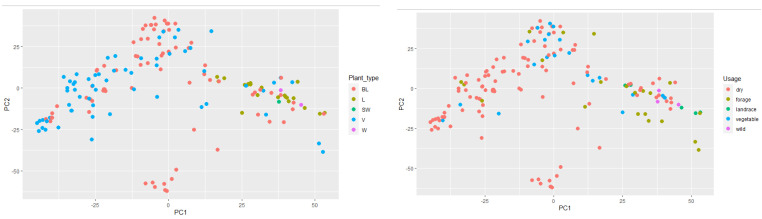
Principal component analysis (PCA) of the broad phenotypic diversity of the investigated pea panel composed by analyzed genotypes, classified by using the DNA extraction data. BL–breeding line; L–landrace; SW–semi-wild; V–variety; W–wild. (**Left**): plant (population) type; (**Right**): variety type by usage.

A Principal Component Analysis (PCA) was also performed on the genotype data and the data for the first two components were related to population type (Figure 6, left) and plant type (Figure 6, right). In the PCA graph grouped by plant type, it is noticeable that landraces and semi-wild and wild materials were concentrated on the cluster’s right side, while breeding lines and varieties were mainly concentrated on the left. This shows a clear morphological group formed from landraces (longer internodes), semi-wild and wild, and the modern bred dry pea varieties.

PCA analysis in relation to plant usage shows and reinforces the structure in the PCA graph by plant type, successfully grouping modern bred varieties (dry and vegetable) whose accessions dominate on the left, while the landrace, forage, and wild types, being clearly distinct phenotypically, are on the right-hand side.

**Figure 7:**
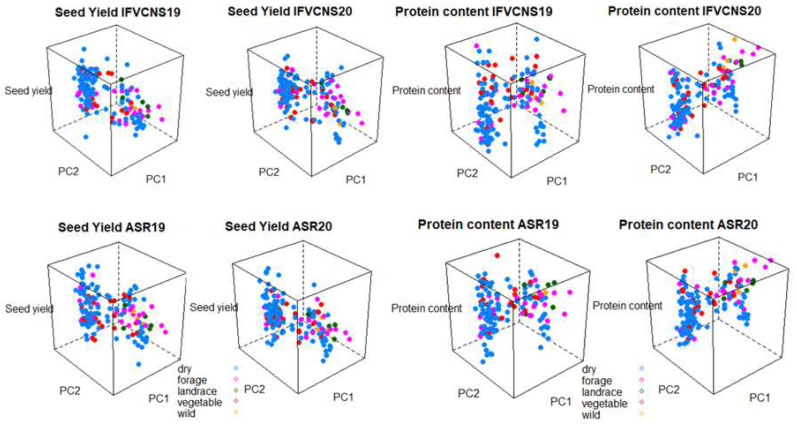
3D graph of the pea seed yield and pea protein content projected on the first two principal components from the PCA of the genotype data for the type of use. (**Left**): seed yield; (**Right**): protein content.

**Figure 8:**
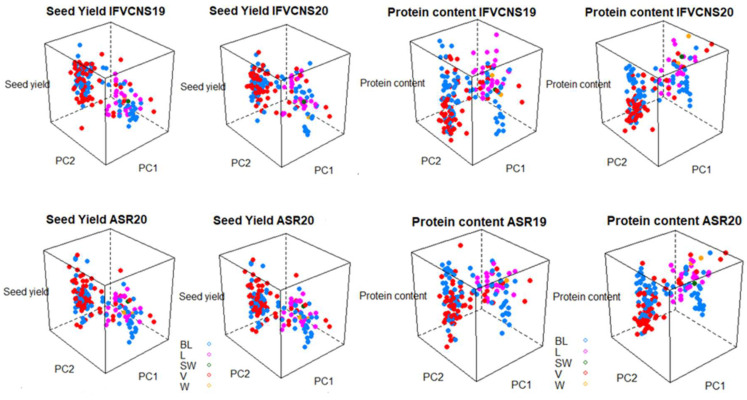
3D graph of the pea seed yield and pea protein content (the first two principal components from the PCA of the genotype data) for the plant type. (**Left**): seed yield; (**Right**): protein content. B–breeding Line; V–variety; SW–semi-wild; W–wild; L–landrace.

The seed yield and protein content was projected on the first two principal components from the PCA of the genotype data to further analyze the phenotypic variance and G*E interaction using 3D graphs (Figure 7 and Figure 8). The interactions between the genotypes of the accessions, the two sites, years, and usage or plant type showed that for seed yield, the dry and vegetable types tended to have the widest range of yields irrespective of year and site, while the forage, landraces, and wild types tended to be intermediary (Figure 7). For protein content, the forage, landraces and wild types were highest, particularly in 2020, at both sites.

In terms of plant type, the varieties and breeding lines had the accessions with the highest seed yield, but also the widest range of yield. Again, the patterns were similar within the same year at the two sites (Figure 8). For protein content, landraces and wild and semi-wild accessions were in the high category, together with a few varieties, particularly in 2020.

## 3. Discussion

High variability in germplasm collection is desirable when it comes to introducing new sources of variation in breeding programs. Evaluation and characterization of inherited agronomic traits can improve the identification of the phenotypic clusters within broad geographic groups [21]. In order to assess the phenotypic variability of a pea germplasm collection, the partially-replicated experimental design used in this work allowed successful estimation of broad-sense heritability (He^2^_B_) using limited seed quantities, significantly reducing the cost of phenotyping [22] a diversity panel containing a large number of lines. Extensive phenotypic variation was present in the diversity panel at both trial sites, illustrated particularly by high coefficients of variation for SY (at IFVCNS and Agro Seed), for TSW (at IFVCNS), and for GPP (at Agro Seed), as well as for SWPP and PL (at IFVCNS) in 2020 and for PH in 2019 and TSW in 2020 (at Agro Seed), while for PH, higher values were recorded in the 2020 season for both trial sites. High heritability of TSW was observed at both IFVCNS and Agro Seed, similar to previous findings [15,19,23,24], showing that this trait is highly genetically controlled. The lowest heritability was recorded for PPP in 2019 and SPP in 2020 for IFVCNS, and for PPP in 2019 and PL in 2020 for Agro Seed, similar to the findings of [19]. Lower heritability for these traits could be due to the significant error variance caused by high environmental influence (particularly with regards to rainfall) and not the narrow genetic variance. Means for PPP, SPP, SWPP, and PH were significantly lower in 2020 than in 2019, which might be the consequence of drought during the flowering period in May 2020 in Serbia (Table S1). The mean for SY was higher in 2020; similar findings were given by [6], where a high amount of precipitation, especially at the flowering stage, positively affected the grain yield of field pea. Means for PPP, SPP, PH, and SY were lower in 2019 in Belgium. In contrast, TSW was higher in 2019 compared to 2020, which is similar to the findings of [25], where lack of moisture in the pod-filling period led to lower number of pods and seeds per plant. This causes the plants to concentrate their nutrients and energy in the weight of the seeds, giving a higher mass of 1000 seeds, but generally lower yields. The coefficient of variation for all traits in the Serbia trial was generally higher in 2020, which is most likely due to uneven rain distribution during the pea growing season (Table S1), which can affect all the elements of the yielding structure, the length of flowering, and the height and lodging of plants.

The phenotypic correlation is conditioned by the relationship among individual traits and the influence of environmental factors [26]. If there is a correlation between two traits, direct selection of one will cause a change in the other. Before initiating any effective selection program, it is necessary to know the association of various traits with each other [27]. The relationship between various traits of peas has been studied previously, but the results were found to vary significantly according to varietal differences and environmental conditions [28,29,30,31]. This study used a very diverse group of pea genotypes and many varieties have a low number of seeds per pod or small grains, therefore diluting the correlation. However, in both trial sites, a positive and significant correlation was found between the traits that represent plant yield (SWPP) with PoL, PPP, SPP, and TSW, similar to previous findings [32,33], as well as a high positive correlation of SY with TSW and SWPP. The high positive correlation between PPP and SPP is similar to the results of [34]. These results indicate that number of pods per plant has a significant influence on yield. The positive correlation of PPP with PH and the negative correlation of GPP with PH, both observed at IFVCNS, is similar to [35], suggesting that the height of the plant could affect the number and size of seeds. Furthermore, the negative correlation between PH and PoL and the positive correlation between PoL and GPP observed at IFVCNS may indicate an indirect negative correlation between height and grains per pod. A significant negative correlation was expressed between TSW with GPP, PPP, and SPP for both trial sites, and GPP with PL (in the IFVCNS trial). SPP and FL were positively correlated only in the Agro Seed trial. PC was significantly negatively correlated with TSW only in the IFVCNS trial, unlike the results of [36], who noted positive correlations between these two traits. Our results for the IFVCNS trial, similar to [37], showed that in pea, the relationship between seed yield and seed weight per plant is always positive, regardless of the environment and the genetic background, which implies that the relationship between seed protein content and seed yield is almost always negative. Correlations between remaining pairs of traits mainly were low and non-significant in both years, which might indicate the presence of nonlinear interaction between traits [38].

Correlation of a single trait between years was analyzed. A low correlation between years might suggest a higher genotype by environment (GxE) interaction [15]. Thus, it can be concluded that traits with lower correlation between 2019 and 2020, such as PPP, SPP, PL, and SWPP, were influenced by environmental factors, while TSW, PoL, GPP, and PH were less affected. Similar findings were observed by [15] and [19], which also detected lower GxE interactions for TSW and higher interactions for SPP. The lowest correlation was observed for FL, which might indicate genotype reaction to agro-ecological conditions (high GxE). This might be influenced by the fact that the varieties, wild accessions, and landraces included in the assessed germplasm collection originated from different climatic conditions. Since the correlations of all examined traits in two years were positive, it can be concluded that the studied set of genotypes did not have strong reactions to different agro-ecological factors.

Despite the absence of clearly separate groups among pea genotypes based on plant type, comparison among groups by different subtypes (forage, dry, and vegetable) indicated their differences in phenotypic traits based on PCA, so deviations in morphological characteristics between groups comparing trial sites can still be visible. Based on the IFVCNS trials, where the grouping of most landraces is visible on the PCA diagram (Figure 5, left), grouping could result from landrace characteristics to have a higher plant height and smaller grains. Clustering can also be seen for some of the varieties and breeding lines. However, both these groups consist of genotypes from different climates and origins and different uses. Further studies, similar to [15] and [39], performed at the molecular level, could show a more detailed grouping of these genotypes. Comparing type by use, in the IFVCNS trial, clustering can be seen mostly for the dry pea subtype, followed by the forage subtype (Figure 6, left). Dry pea varieties are characterized by small number of seeds per pod, large grains, and about 20–25% protein content [40,41], while forage peas are mainly characterized by small grains, indeterminate type, and 17–24% protein content [41,42]. Similar clustering can be seen in the Agro Seed trial, with a less visible structure. Wild and landrace types are clustered together (on the right).

There were marked phenotypic differences between the wild peas (climbing, low lodging resistance, indeterminate growth) and commercial pea varieties (dry pea, forage). However, there was significant variation in the traits themselves, which was confirmed by analysis of variance. In contrast, when the genotypes were analyzed by the population type, no apparent structure of phenotypic data was present because, within groups (such as breeding lines, varieties, landraces), there were variations resulting from different subtype representations (such as dry pea, vegetable pea, forage pea) within each of them.

Based on the hierarchical clustering, there was evidently a difference between genotypes. This can be explained by the fact that both pea varieties and pea breeding lines can be classified into vegetable peas, dry peas for feed and food, and forage peas (grown primarily for forage) [42]. Landraces have distinctive characteristics arising from development and adaptation over time to conditions of a localized geographic region, and typically display greater genetic diversity than types subjected to formal breeding practices [43].

Higher correlations for the IFVCNS trials, compared to the Agro Seed trials, as calculated by the Mantel test, may partially be because the analyzed pea panel consisted of 64 accessions with a Serbian provenance. If they were adapted to conditions in Serbia more than other accessions, this may be reflected in the overall genetic makeup, meaning that these accessions would likely be genetically more similar due to adaptation to conditions in this specific region. In contrast, accessions from a broader range of geographic and ecologically distinct regions will be more genetically diverse.

The fact that landraces and semi-wild, and wild material were separated from most breeding lines and varieties (Figure 6, Left), and that forage types were separated from garden and dry peas (Figure 6, Right) confirms that there is a decrease in similarity between the genotypes, which can be explained by their different purposes.

The 3D graphs (Figure 7 and Figure 8) show that the patterns of variation in seed yield and protein content were remarkably similar at the two sites and relatively consistent between years. This was the case regardless of whether the accessories were grouped according to use or plant type; this confirms the adaptability of peas to different agroclimatic factors.

## 4. Materials and Methods

### 4.1. Plant Material

The study was conducted on 165 pea genotypes (Table S4) during two vegetation seasons—2019 and 2020—In the experimental field of the Institute of Field and Vegetable Crops (IFVCNS), Novi Sad, Rimski šančevi, Serbia (45°20′ N, 19°51′ E), and in the experimental field of the Agro Seed Research company, Kessenich, Belgium (51°08′ N, 5°48′ E). Studied genotypes encompassed 78 breeding lines, 59 varieties, 25 landraces and two wild and one semi-wild accession. The analyzed panel was set up to represent relevant European pea diversity and also included genotypes from Asia, Australia, and North America (USA). Genotypes used in the experiment were grouped based on variety by type into several groups: dry, forage, and vegetable. All material was kindly provided from germplasm collections from four institutions: Institute of Field and Vegetable Crops, Novi Sad, Serbia (IFVCNS), Agro Seed Research, Kessenich, Belgium (ASR), Institute of Biological, Environmental and Rural Sciences, Aberystwyth, United Kingdom (IBERS), and NorthGen Genetic Resource Center, Alnarp, Sweden (NorthGen).

### 4.2. Experimental Design and Phenotyping

The experiment was set up according to an experimental plan with partial repetitions similar to [44,45,46,47] in a row-column system with four experimental blocks. The trial was set up using augmented block design to minimize land and labor costs while still controlling for sources of variation. It has been shown that augmented designs are especially useful for assessing genotype effects practically and efficiently [17]. The size of an individual plot was 5 m^2^. The distance between the rows in the plot was 0.2 m so each plot had five rows, with a distance between the plots of 0.9 m. Of the total 165 genotypes, 146 were sown in one replicate, while 19 genotypes were presented in four replicates, so the total number of plots was 222. The usual pre-sowing preparation was done and the sowing depth was 2 cm, with a plant density of 80 plants/m^2^. After sowing, standard field practices were applied. Sowing was done in early March and the harvest was in late July in both years and at both trial sites.

Seed yield components data were collected from ten randomly selected plants, avoiding marginal rows. The analyzed traits were grains per pod (GPP), pods per plant (PPP), seeds per plant (SPP), flowering duration (FD), plant height (PH), plot lodging (PL), pod length (PoL), thousand seed weight (TSW), seed weight per plant (SWPP), seed yield (SY), and protein content (PC). Plot lodging was determined by measuring the height of the plot in full flowering and again before harvest. The thousand seed weight were determined according to a method in which 100 grains from each subplot were weighed using an analytical scale and the obtained result multiplied by 10 [48]. Protein content was determined using Fourier-transformed near infra-red spectroscopy (FT-NIRS) via an FT-NIR analyzer (Antaris Thermo Fisher Scientific, Waltham, MA, USA) [49,50].

### 4.3. Meteorological Conditions

The Novi Sad area is characterized by a moderate continental climate, with an average annual temperature of 11 °C and 122 sunny days per year [51]. The meteorological conditions in Novi Sad during the vegetation season of pea in 2019 and 2020 and long-term averages are presented in Table S1. The average temperature in 2019 was higher by 1.5 °C compared to the long-term average, while in 2020, it was higher by 1.3 °C. The precipitation in 2019 was higher by 5.7 mm, whereas the precipitation in 2020 was 14.3 mm higher than the long-term average, with uneven distribution of precipitation per month.

The Kessenich area has a more continental climate compared to the rest of Belgium because it is less influenced by the Atlantic Ocean. Precipitation is frequent, but not particularly abundant. The annual average temperature is 10 °C [52]. The meteorological conditions in Kessenich during the vegetation season of pea in 2019 and 2020 and the long-term average are presented in Table S2. The average temperature in 2019 was higher by 1.06 °C compared to the long-term average, while in 2020, it was higher by 1.15 °C. The precipitation in both years was slightly higher than the long-term average (0.6 and 0.8 mm, respectively), with even distribution per month.

### 4.4. Statistical Analysis

The spatial arrangement of plots in the field was used to assign row and column coordinates in each trial. For each trait, the best linear unbiased predictor (BLUP) was obtained for each genotype and year, using the spatial model as described by [53]. The variance components, estimated following [20], were used to assess the broad-sense heritability of each trait:

He^2^_B_ = (σ^2^_g_/σ^2^_p_) × 100 (1)

where: 

σ^2^_p_ = σ^2^_e_ + σ^2^_g_: Phenotypic variance;

σ^2^_g_: Genotypic variance;

σ^2^_e_: Error variance.

A principal component analysis (PCA) was conducted on normalized data using Minitab 17 software Trial version (Minitab Inc., Pennsylvania State University), taking into account sample plant type and variety type by usage. PCA results were graphically summarized in a biplot. Mixed model analysis, described by [54], was performed using Progeno 3.6.24 software (Progeno BV Company, Ghent University, Ghent, Belgium) [55]. Descriptive statistics and Pearson’s correlation coefficients between BLUPs for all quantitative traits were analyzed in XLStat according to [56], and analysis of variance was performed using Minitab 17 software Trial version (Minitab Inc., Pennsylvania State University, State College, PA, USA) [57].

### 4.5. DNA Extraction and Genotyping

DNA was extracted from 50 mg leaf sections of each accession using the QIAGEN DNAEasy 96 plant kit (QIAGEN, Manchester, UK). DNA samples were genotyped by NEOGEN EUROPE LTD (GENESEEK EUROPE), Auchincruive, Ayr, Scotland. The 13.2K GenoPea array (Illumina), was first described by [58]. The data were imported into the R program “argyle”, where initial assessment and quality control took place [59]. A total of 586 markers were removed due to low signal (1) and more than 20 no-calls in a sample (585). Based on the DNA extraction results, the hierarchical clustering, principal component analysis (PCA), and Mantel tests were performed using R (R Core Team 2020). For the Mantel Test, the R package “ade4” was used. The distance matrices were generated using the “Euclidian” method. The fan plot of the hierarchical clustering was done using the “ape” package. The PCA analysis was carried out using the prcomp command in base R [60].

## 5. Conclusions

Examination of phenotypic diversity of pea genotypes of different origins indicated considerable variation for a range of traits. The broad-sense heritability estimates were moderate to high for all examined traits, indicating the major influence of genetic factors. Inconsistent heritability between years and between sites for certain traits indicated their higher sensitivity to environmental conditions. Based on this work, it could be concluded that selection of traits with high and consistent heritability would be most valuable to breeders, including stable traits that effect seed weight per plant such as pod length and thousand seed weight. Moderate, consistent heritability with a long-lasting effect on seed weight per plant was observed for the number of seeds per plant, so direct selection of this trait could also be effective. Breeding for increased seed protein content is hampered by the negative correlation between protein content and yield. Therefore, increasing protein production can be done by increasing the area under protein crops or by improving protein quality over protein content, given that difference in protein content can be the result of many different environmental factors. The seed yield is a less challenging breeding target than protein content; therefore, high yields have been achieved among some of the cultivated accessions. Given the lack of the strong influence of GxE, confirmed by positive correlations between two years, this gene pool provides numerous possibilities as a starting point for future pea varieties adaptable to different agroclimatic conditions. Traits such as the number of grains per pod, plant height, pod length, thousand seed weight, seed yield, and protein content showed highly significant variations among the tested groups of genotypes. Knowledge of these variations could be used in further plant selection programs.

Given the proven and evident differences between the analyzed genotypes based on type and use, and their great adaptability to tested conditions, it can be concluded that pea is a very adaptable plant species with untapped production potential.

The results of this study should contribute to a better knowledge of variability and seed yield stability of pea genotypes used in Europe for future production and breeding. Obtained phenotypic results could improve pea breeding by developing new cultivars carrying favorable traits to attain more sustainable production and higher yields. In conclusion, this work should promote the broader use of pea as a grain legume within diverse agricultural systems to provide multiple beneficial advantages, in line with the principles of sustainability.

## Figures and Tables

**Table 1:** Descriptive statistics for eleven traits evaluated in pea genotypes ats IFVCNS, Serbia.

Trait	2019							2020							
	Min	Max	Mean	SD	CV (%)	SE	He^2^_B_	Min	Max	Mean	SD	CV (%)	SE	He^2^_B_	*p* Values (*t* Test)
**GPP**	3.38	5.49	4.34	0.42	10	0.03	47.65	2.17	7.62	4.66	0.85	18	0.07	74.22	**
**PPP**	8.78	21.4	13.36	1.78	13	0.14	43.57	6.41	13.9	9.4	1.56	17	0.12	55.34	**
**SPP**	35.4	93.91	57.86	10.27	18	0.8	52.66	25.58	68.7	43.5	8.35	19	0.65	54.24	**
**FD**	12	38.21	22.85	4.78	21	0.37	87.52	9.40	41.7	22.06	4.64	21	0.36	79.32	0.06
**PH**	57.6	145.6	99.8	15.58	16	1.22	80.66	27.21	103	71.04	15.71	22	1.22	85.19	**
**PL**	0.42	0.88	0.69	0.10	15	0.01	54.56	0.32	0.93	0.68	0.15	22	0.01	82.03	0.11
**PoL**	3.16	8.93	5.76	0.90	16	0.07	91.44	4.04	9.91	5.97	0.84	14	0.07	93.12	**
**TSW**	37.2	315.8	174.7	53.48	31	4.18	96.60	40.54	307	187.5	54.92	30	4.31	97.70	**
**SWPP**	47	169.8	95.18	20.91	22	1.63	54.50	37.63	122	70.87	16.01	23	1.25	59.53	**
**SY**	305	2957	1555	0.42	42	51.1	86.89	2.17	3439	1813	0.85	36	50.2	86.88	**
**PC**	23.9	30.91	26.51	1.78	5	0.11	82.59	6.41	31.9	27.97	1.56	5	0.12	85.12	**

GPP—grains per pod (#); PPP—pods per plant (#); SPP—seeds per plant (#); FD—flowering duration (days); PH—plant height (cm); PL—plot lodging; PoL—pod length (cm); TSW—thousand seed weight (g); SWPP—seed weight per plant (g); SY—seed yield (g/plot); PC—protein content (%); SD—standard deviation; CV—coefficient of variation (%); SE—standard error; He^2^_B_—broad-sense heritability (%); *p*-value (*t* test done for two-year performance measurement results). Significant at *p* ≤ 0.01 (**).

**Table 2:** Descriptive statistics for eleven traits evaluated in pea genotypes at Agro Seed, Belgium.

	2019							2020							
	Min	Max	Mean	SD	CV (%)	SE	He^2^_B_	Min	Max	Mean	SD	CV (%)	SE	He^2^_B_	*p* Values (*t* Test)
**GPP**	1.43	13.29	3.72	1.02	27	0.11	69.55	1.09	13.5	3.7	1.05	28	0.08	70.59	0.12
**PPP**	4.81	8.47	6.07	0.57	9	0.00	27.58	7.43	18.2	9.98	1.49	15	0.12	42.03	**
**SPP**	13.21	38.20	22.13	4.18	19	0.32	45.01	26.5	76.9	41.97	6.74	16	42	42.72	**
**FD**	9.58	18.27	13.69	1.37	10	0.11	55.10	11.1	26	16.18	2.79	17	0.22	65.86	**
**PH**	48.52	150	84.78	22.20	26	85	87.11	55.5	154	92.25	19.2	21	1.51	73.13	**
**PL**	0.17	0.77	0.41	0.15	37	0.00	67.39	0.33	0.77	0.49	0.07	14	0.01	30.95	**
**PoL**	1.06	7.32	4.96	0.75	15	0.13	79.79	3.67	7.72	5.39	0.66	12	0.05	79.10	**
**TSW**	58.91	341	201.8	50.50	25	3.9	89.50	63.2	284	172.64	47.2	27	3.67	83.35	**
**SWPP**	3.05	6.74	4.61	0.65	14	0.11	33.65	4.52	13.7	7.79	1.64	21	0.13	49.52	**
**SY**	418	2180	1277	399	31	31	67.74	672	3349	1713.9	592	35	46.1	70.20	**
**PC**	23.1	30.4	26.94	1.22	5	0.12	84.24	21.7	32.3	27.48	1.74	6	0.14	88.55	**

GPP—grains per pod (#); PPP—pods per plant (#); SPP—seeds per plant (#); FD—flowering duration (days); PH—plant height (cm); PL—plot lodging; PoL—pod length (cm); TSW—thousand seed weight (g); SWPP—seed weight per plant (g); SY—seed yield (g/plot); PC—protein content (%); SD—standard deviation; CV—coefficient of variation (%); SE—standard error; He^2^_B_—broad-sense heritability (%); *p*-value (*t* test done for two-year performance measurement results). Significant at *p* ≤ 0.01 (**).

**Table 3:** Correlation coefficients between pea yield components at IFVCNS, Serbia.

**Variables**	**GPP**	**PPP**	**SPP**	**FD**	**PH**	**PL**	**PoL**	**TSW**	**SWPP**	**SY**	**PC**
**GPP**											
**PPP**	−0.12										
**SPP**	0.41 **	0.76 **									
**FD**	−0.18 *	0.16 *	0.02								
**PH**	−0.33 **	0.23 **	0.07	0.13 *							
**PL**	−0.41 **	0.14	−0.21 **	−0.06	−0.07						
**PoL**	0.41 **	−0.42 **	−0.18 *	−0.09	−0.25 **	−0.21 **					
**TSW**	−0.19 **	−0.42 **	−0.6 *	0.08	−0.04	0.16 **	0.56 **				
**SWPP**	0.22 **	0.17 *	0.23 **	0.13	−0.08	−0.03	0.56 **	0.55 **			
**SY**	−0.02	−0.1	−0.13	0.18 *	−0.2	0.21 *	0.29 **	0.58 **	0.64 **		
**PC**	0	0.11	0.14	−0.1	0.14	−0.16	−0.22	−0.44 *	−0.46 **	−0.7 **	

GPP—grains per pod; PPP—pods per plant; SPP—seeds per plant; FD—flowering duration; PH—plant height; PL—plot lodging; PoL—pod length; TSW—thousand seed weight; SWPP—seed weight per plant; SY—seed yield; PC—protein content. Significant at *p* ≤ 0.05 (*), *p* ≤ 0.01 (**).

**Table 4:** Correlation coefficients between pea yield components at Agro Seed, Belgium.

Variables	GPP	PPP	SPP	FD	PH	PL	PoL	TSW	SWPP	SY	PC
**GPP**											
**PPP**	−0.05										
**SPP**	0.21 **	0.75 **									
**FD**	0	0.41 **	0.29 **								
**PH**	0.1	0.46 **	0.30 **	0.44 **							
**PL**	0.03	−0.09	−0.06	−0.01	−0.12						
**PoL**	−0.02	−0.20 *	−0.06	−0.20 *	−0.12	0.04					
**TSW**	−0.17 *	−0.26 **	−0.37 **	−0.08	0.08	0.29 **	0.43 **				
**SWPP**	0.05	0.36 **	0.40 **	0.05	0.21 **	0.18 *	0.45 **	0.51 **			
**SY**	0.01	−0.04	0.07	−0.05	−0.12	0.5	0.23 **	0.52 **	0.64 **		
**PC**	−0.1	0.1	0.23 **	0.01	−0.1	0.02	0.08	−0.2	0.07	0.14 **	

GPP—grains per pod; PPP—pods per plant; SPP—seeds per plant; FD—flowering duration; PH—plant height; PL—plot lodging; PoL—pod length; TSW—thousand seed weight; SWPP—seed weight per plant; SY—seed yield; PC—protein content. Significant at *p* ≤ 0.05 (*), *p* ≤ 0.01 (**).

**Table 5:** Analysis of variance for data collected on pea genotypes tested on both sites.

	IFVCNS, Serbia				Agro Seed, Belgium			
Trait	2019		2020		2019		2020	
	F	*p*-Value	F	*p*-Value	F	*p*-Value	F	*p*-Value
**GPP**	33.171	0.0122 *	53.086	0.0005 **	0.45	0.772	0.55	0.696
**PPP**	17.281	0.1463	0.6242	0.6459	1.10	0.357	1.44	0.222
**SPP**	30.741	0.018 *	12.562	0.2895	2.01	0.096	0.16	0.957
**FD**	14.681	0.2134	0.3419	0.8494	0.27	0.894	1.58	0.183
**PH**	79.438	0.000 **	11.588	0.000 *	14.28	0.000 **	14.32	0.000 **
**PL**	0.6091	0.6566	17.638	0.1387	3.42	0.010	2.05	0.089 *
**PoL**	49.446	0.0009 **	31.052	0.0171 *	1.66	0.162	3.16	0.016 *
**TSW**	37.281	0.0063 *	3.375	0.0111 *	3.85	0.005 **	2.57	0.040 *
**SWPP**	30.949	0.017 *	16.254	0.170	1.51	0.201	0.47	0.761
**SY**	10.22	0.000 **	4	0.009 *	1.97	0.101	3.26	0.013 *
**PC**	4.87	0.001 **	9.62	0.000 **	2.50	0.045	6.1	0.000 **

GPP—grains per pod; PPP—pods per plant; SPP—seeds per plant; FD—flowering duration; PH—plant height; PL—plot lodging; PoL—pod length; TSW—thousand seed weight; SWPP—seed weight per plant; SY—seed yield; PC—protein content. Significant at *p* ≤ 0.05 (*), *p* ≤ 0.01 (**).

**Table 6:** Mantel test results for correlations between the genotype distance matrix and the phenotype distance matrices at the IFVCNS and ASR sites in 2019 and 2020.

Mantel Test	IFVCNS19	IFVCNS20	ASR19	ASR20
Correlation (r)	0.238	0.240	0.120	0.110
Probability	1 × 10^−4^	1 × 10^−4^	2 × 10^−4^	6 × 10^−4^

## Data Availability

The data presented in this study are available on request from the corresponding author. The data are not publicly available due to the ongoing project.

## Supplementary Materials

The following supporting information can be downloaded at: https://www.mdpi.com/article/10.3390/plants11101321/s1, Table S1: Meteorological conditions in Novi Sad, Serbia during the vegetation seasons of pea in 2019 and 2020, and long-term average. Table S2: Meteorological conditions in Kessenich, Belgium during the vegetation seasons of pea in 2019 and 2020, and long-term average. Table S3: Pearson’s correlation coefficient ® for analyzed traits between two years (2019 and 2020). Table S4: The list of pea genotypes analyzed in the study.
