# Supplementary material for: Phenotypic and Genotypic Characterization and Correlation Analysis of Pea (Pisum sativum L.) Diversity Panel
Source: Plants (Basel). 2022 May 16;11(10):1321. doi: 10.3390/plants11101321 (PMC9146737; doi:10.3390/plants11101321)
Supplement: Supplementary file 1 [file plants-11-01321-s001.zip › plants-1653240-supplementary.pdf]

Table S1: Meteorological conditions in Novi Sad, Serbia during the vegetation seasons of pea in 2019 and 2020, and long-term average temperatures

Temperature (°C)				Precipitation (mm)			
Month	Year		Long-term average	Month	Year		Long-term average
	2019	2020			2019	2020	
I	0.1	0.6	0.5	I	42	23	40
II	4.9	7.2	1.7	II	16	46	33
III	10.1	8.8	6.4	III	18	55	42
IV	14.6	12.6	11.9	IV	54	12	48
V	14.9	16.1	17.2	V	147	48	73
VI	22.8	20.9	20.2	VI	64	163	23
VII	22.7	22.8	22.1	VII	22	76	64
**Average**	**12.9**	**12.7**	**11.4**	**Average**	**51.8**	**60.4**	**46.1**

Table S2: Meteorological conditions in Kessenich, Belgium during the vegetation seasons of pea in 2019 and 2020, and long-term average temperatures

Temperature (°C)				Precipitation (mm)			
Month	Year		Long-term average	Month	Year		Long-term average
	2019	2020			2019	2020	
I	1.6	4.5	2.1	I	0.35	0.35	0.28
II	5.3	6.3	2.8	II	0.29	0.48	0.29
III	7.8	6.1	5.8	III	0.35	0.35	0.29
IV	10.7	11.6	9.9	IV	0.33	0.35	0.27
V	12.2	13.4	13.7	V	0.35	0.35	0.29
VI	19.8	17.9	17.1	VI	0.44	0.35	0.30
VII	20.5	18.5	19.1	VII	0.35	0.36	0.30
**Average**	**11.1**	**11.2**	**10.1**	**Average**	**0.35**	**0.37**	**0.29**

Table S3. Pearson's correlation coefficient (r) for analyzed traits between two years (2019 and 2020)

Trait	Serbia	Belgium
	r	r
**GPP**	0.60***	0,98
**PPP**	0.44***	0,40***
**SPP**	0.45***	0,28***
**FL**	0.13	0,10***
**PH**	0.77***	0,82***
**PL**	0.46***	0,68***
**PoL**	0.75***	0,61***
**TSW**	0.92***	0,80***

**SWPP**	0.48***	0,42***
**SY**	0,74***	0,73***
**PC**	0,27***	0,17***

Significant at p ≤ 0.001(***)

Table S4: The list of pea genotypes analysed in the study

Eucleg no	Accession name	Accession Number	Population type *	Country of origin	Variety type by usage	Gen collection**
**EUC_PS_001**	00-10	00-10	BL	North America	Dry	ASR
**EUC_PS_002**	00-11	00-11	BL	North America	Dry	ASR
**EUC_PS_003**	00-2060	00-2060	BL	North America	Dry	ASR
**EUC_PS_004**	00-2061	00-2061	BL	North America	Garden	ASR
**EUC_PS_005**	00-2062	00-2062	BL	North America	Dry	ASR
**EUC_PS_006**	00-2063	00-2063	BL	North America	Garden	ASR
**EUC_PS_007**	00-2064	00-2064	BL	North America	Dry	ASR
**EUC_PS_008**	00-2066	00-2066	BL	North America	Garden	ASR
**EUC_PS_009**	00-2067	00-2067	BL	North America	Dry	ASR
**EUC_PS_010**	00-2068	00-2068	BL	North America	Dry	ASR
**EUC_PS_011**	00-2069	00-2069	BL	North America	Dry	ASR
**EUC_PS_012**	00-2071-2	00-2071-2	BL	North America	Garden	ASR
**EUC_PS_013**	00-2072	00-2072	BL	North America	Dry	ASR
**EUC_PS_014**	00-2073	00-2073	BL	North America	Garden	ASR
**EUC_PS_015**	00-2084	00-2084	BL	North America	Dry	ASR
**EUC_PS_016**	00-2086	00-2086	BL	North America	Garden	ASR
**EUC_PS_017**	00-2087	00-2087	BL	North America	Garden	ASR
**EUC_PS_018**	00-2091	00-2091	BL	North America	Dry	ASR
**EUC_PS_019**	00-2100	00-2100	BL	North America	Dry	ASR
**EUC_PS_020**	00-2103	00-2103	BL	North America	Dry	ASR
**EUC_PS_021**	00-2106	00-2106	BL	North America	Dry	ASR
**EUC_PS_022**	00-2122	00-2122	BL	North America	Dry	ASR
**EUC_PS_023**	00-2177	00-2177	BL	North America	Dry	ASR

**EUC_PS_024**	00-7	00-7	BL	North America	Garden	ASR
**EUC_PS_025**	03PP054.46	03PP054.46	V	Australia	Dry	ASR
**EUC_PS_026**	171-11001	171-11001	BL	North America	Forage	ASR
**EUC_PS_027**	AP18	AP18	V	North America	Forage	ASR
**EUC_PS_028**	AP2	AP2	V	North America	Forage	ASR
**EUC_PS_029**	Ariel	Ariel	V	New Zealand	Dry	ASR
**EUC_PS_030**	Banner	Banner	V	North America	Dry	ASR
**EUC_PS_031**	Columbia	Columbia	V	North America	Dry	ASR
**EUC_PS_032**	Courier	Courier	V	North America	Dry	ASR
**EUC_PS_033**	Cruiser	Cruiser	V	North America	Dry	ASR
**EUC_PS_034**	Ginny	Ginny	V	North America	Dry	ASR
**EUC_PS_035**	H3-2	H3-2	V	North America	Forage	ASR
**EUC_PS_036**	hyline	hyline	V	North America	Dry	ASR
**EUC_PS_037**	icicle	icicle	V	North America	Forage	ASR
**EUC_PS_038**	Journey	Journey	V	North America	Forage	ASR
**EUC_PS_039**	Koyote	Koyote	V	North America	Winter dry	ASR
**EUC_PS_040**	Monarch	Monarch	V	North America	Dry	ASR
**EUC_PS_041**	Pacifica	Pacifica	V	North America	Dry	ASR
**EUC_PS_042**	PRL 6254	PRL 6254	V	New Zealand	Dry	ASR
**EUC_PS_043**	Pro 6243	Pro 6243	V	North America	Dry	ASR
**EUC_PS_044**	Pro 7123	Pro 7123	V	North America	Dry	ASR
**EUC_PS_045**	Pro 7127	Pro 7127	V	North America	Dry	ASR
**EUC_PS_046**	Pro 7405	Pro 7405	V	North America	Dry	ASR
**EUC_PS_047**	Pro 7410	Pro 7410	V	North America	Dry	ASR
**EUC_PS_048**	Pro 822	Pro 822	V	North America	Dry	ASR
**EUC_PS_049**	Pro101-7133	Pro101-7133	V	North America	Dry	ASR
**EUC_PS_050**	Whero	Whero	V	North America	Dry	ASR
**EUC_PS_051**	Whistler	Whistler	V	North America	Winter forage	ASR
**EUC_PS_052**	Yarrum	Yarrum	V	Australia	Dry	ASR
**EUC_PS_053**	ASR 4064	ASR 4064	V	Belgium	Garden	ASR

**EUC_PS_054**	ASR 4027	ASR 4027	V	Belgium	Garden	ASR
**EUC_PS_055**	ASR 4139	ASR 4139	V	Belgium	Garden	ASR
**EUC_PS_056**	ASR 4150	ASR 4150	V	Belgium	Garden	ASR
**EUC_PS_057**	ASR 4134	ASR 4134	V	Belgium	Garden	ASR
**EUC_PS_058**	JI 2713	JI2713	SW	Russia	Wild	IBERS
**EUC_PS_059**	JI 2546	JI2546	W	Georgia	Wild	IBERS
**EUC_PS_060**	JI 201	JI0201	W	Italy	Wild	IBERS
**EUC_PS_061**	JI 1346	JI1346	L	Mongolia	Landrace	IBERS
**EUC_PS_062**	JI 2545	JI2545	L	Netherlands	Landrace	IBERS
**EUC_PS_063**	JI 3541	JI3541	BL	India	Sugar snap	IBERS
**EUC_PS_065**	JI 1478	JI1478	L	Afganistan	Landrace	IBERS
**EUC_PS_066**	JI 778	JI0778	BL	North America	Dry	IBERS
**EUC_PS_067**	JI 3022	JI3022	GS	Russia	Dry	IBERS
**EUC_PS_068**	JI 1482	JI1482	L	Afganistan	Landrace	IBERS
**EUC_PS_069**	JI 1124	JI1124	L	Nepal	Landrace	IBERS
**EUC_PS_110**	Campus	Campus	V	Denmark	Dry	IBERS
**EUC_PS_111**	Kareni	Kareni	V	France	Dry	IBERS
**EUC_PS_112**	Mascara	Mascara	V	Denmark	Dry	IBERS
**EUC_PS_113**	Sakura	Sakura	V	United Kingdom	Marrowfa t	IBERS
**EUC_PS_114**	JULITA	NGB9927	L	Sweden	Sugar pea	NorthGen
**EUC_PS_117**	BLÅ ÄRTER 1	NGB11727	L	Sweden	Dry	NorthGen
**EUC_PS_118**	BLÅÄRT 2	NGB11735	L	Sweden	Dry	NorthGen
**EUC_PS_120**	VIDEMOSEÆRT	NGB11759	L	Denmark	Garden	NorthGen
**EUC_PS_121**	GOTLÄNDSK BLÅÄRT	NGB11760	L	Sweden	Dry	NorthGen
**EUC_PS_126**	SKÅNSK GRÅÄRT	NGB14155	L	Sweden	Dry	NorthGen
**EUC_PS_129**	BOHUSÄRT	NGB14637	L	Sweden	Dry	NorthGen
**EUC_PS_130**	SÖRMLÄNDSK BÖNÄRT	NGB14640	L	Sweden	Dry	NorthGen
**EUC_PS_131**	LIT	NGB14642	L	Sweden	Dry	NorthGen
**EUC_PS_145**	ALFTA	NGB17867	L	Sweden	Dry/garde n	NorthGen
**EUC_PS_146**	VÄSE	NGB17868	L	Sweden	Dry	NorthGen
**EUC_PS_147**	VISINGSÖ	NGB17870	L	Sweden	Dry	NorthGen
**EUC_PS_148**	Tjörn	NGB18053	L	Sweden	Dry	NorthGen
**EUC_PS_153**	SPARLÖSA GRÅÄRT	NGB20190	L	Sweden	Dry	NorthGen
**EUC_PS_154**	HISINGS GRÅÄRT	NGB20192	L	Sweden	Dry	NorthGen
**EUC_PS_155**	TJÖRN från SESAM	NGB21238	L	Sweden	Garden	NorthGen
**EUC_PS_156**	RABER	NGB22830	L	Sweden	Garden	NorthGen

**EUC_PS_162**	WBH 1304	NGB101304	BL	Sweden	Dry	NorthGen
**EUC_PS_163**	Marieholm	NGB101818	L	Sweden	Dry	NorthGen
**EUC_PS_165**	WBH 1846	NGB101846	L	Greece	Garden	NorthGen
**EUC_PS_169**	Smolenskij 812	NGB101987	BL	Russia	Garden	NorthGen
**EUC_PS_170**	Brioärt	NGB101997	V	Sweden	Forage	NorthGen
**EUC_PS_172**	Gråärt	NGB103590	L	Sweden	Dry	NorthGen
**EUC_PS_176**	G32	G32	BL	Serbia	Dry	IFVCNS
**EUC_PS_177**	G28	G28	BL	Serbia	Dry	IFVCNS
**EUC_PS_181**	G12	G12	BL	Serbia	Dry	IFVCNS
**EUC_PS_182**	TR	TR	V	Serbia	Forage/dry	IFVCNS
**EUC_PS_183**	JZ	JZ	V	Serbia	Dry	IFVCNS
**EUC_PS_184**	PR	PR	V	Serbia	Dry	IFVCNS
**EUC_PS_185**	L - 15/4	L-15/4	BL	Serbia	Forage/dry	IFVCNS
**EUC_PS_186**	JV	JV	V	Serbia	Dry	IFVCNS
**EUC_PS_188**	SEP4	SEP4	BL	Serbia	Dry	IFVCNS
**EUC_PS_189**	SEP5	SEP5	BL	Serbia	Dry	IFVCNS
**EUC_PS_190**	SEP8	SEP8	BL	Serbia	Dry	IFVCNS
**EUC_PS_191**	SEP9	SEP9	BL	Serbia	Dry	IFVCNS
**EUC_PS_192**	SEP10	SEP10	BL	Serbia	Dry	IFVCNS
**EUC_PS_193**	SEP11	SEP11	BL	Serbia	Dry	IFVCNS
**EUC_PS_194**	SEP14	SEP14	BL	Serbia	Dry	IFVCNS
**EUC_PS_196**	G47	G47	BL	Serbia	Dry	IFVCNS
**EUC_PS_198**	A7Z	A7Z	BL	Serbia	Dry	IFVCNS
**EUC_PS_199**	A15 Z	A15Z	BL	Serbia	Dry	IFVCNS
**EUC_PS_200**	K1- 18	K1-18	BL	Serbia	Dry	IFVCNS
**EUC_PS_201**	K2- 18	K2-18	BL	Serbia	Dry	IFVCNS
**EUC_PS_202**	G42	G42	BL	Serbia	Forage/dry	IFVCNS
**EUC_PS_203**	G14	G14	BL	Serbia	Forage/dry	IFVCNS
**EUC_PS_206**	G49	G49	BL	Serbia	Dry	IFVCNS
**EUC_PS_207**	PIS 054	PIS054	BL	Serbia	Forage	IFVCNS
**EUC_PS_208**	PIS 179	PIS179	BL	Serbia	Forage	IFVCNS
**EUC_PS_209**	PIS 183	PIS183	BL	Serbia	Forage/dry	IFVCNS
**EUC_PS_210**	PIS 184	PIS184	BL	Serbia	Forage	IFVCNS
**EUC_PS_211**	PIS 067	PIS067	BL	Serbia	Forage	IFVCNS
**EUC_PS_212**	HR 2	HR2	V	Serbia	Forage	IFVCNS
**EUC_PS_213**	KZ5	KZ5	BL	Serbia	Dry	IFVCNS

**EUC_PS_214**	KZ6	KZ6	BL	Serbia	Dry	IFVCNS
**EUC_PS_215**	G - K	G-K	BL	Serbia	Dry	IFVCNS
**EUC_PS_216**	G - Dž	G-Dž	BL	Serbia	Dry	IFVCNS
**EUC_PS_217**	G - F	G-F	BL	Serbia	Dry	IFVCNS
**EUC_PS_218**	K - TM	K-TM	BL	Serbia	Dry	IFVCNS
**EUC_PS_219**	K - MPR	K-MPR	BL	Serbia	Dry	IFVCNS
**EUC_PS_220**	Ob TR	ObTR	BL	Serbia	Forage/dry	IFVCNS
**EUC_PS_221**	Ob KR/2	ObKR/2	BL	Serbia	Dry	IFVCNS
**EUC_PS_222**	Ob K2/1	ObK2/1	BL	Serbia	Dry	IFVCNS
**EUC_PS_223**	Ob K3/1	ObK3/1	BL	Serbia	Dry	IFVCNS
**EUC_PS_224**	A14Z	A14Z	BL	Serbia	Dry	IFVCNS
**EUC_PS_225**	A2Z	A2Z	BL	Serbia	Dry	IFVCNS
**EUC_PS_226**	A8Z	A8Z	BL	Serbia	Dry	IFVCNS
**EUC_PS_227**	G-ČA-O	G-ČA-O	BL	Serbia	Dry	IFVCNS
**EUC_PS_228**	SEP140	SEP140	BL	Serbia	Dry	IFVCNS
**EUC_PS_229**	ObK1/1	ObK1/1	BL	Serbia	Dry	IFVCNS
**EUC_PS_230**	PI - 1	PI-1	V	Serbia	Forage	IFVCNS
**EUC_PS_231**	Ob L2	ObL2	BL	Serbia	Dry	IFVCNS
**EUC_PS_232**	Ob L3	ObL3	BL	Serbia	Dry	IFVCNS
**EUC_PS_233**	Ob L6	ObL6	BL	Serbia	Dry	IFVCNS
**EUC_PS_234**	PIS178	PIS178	BL	Serbia	Forage	IFVCNS
**EUC_PS_241**	Aragorn	Aragorn	V	North America	Dry	ASR
**EUC_PS_242**	Flex	Flex	V	North America	Forage	ASR
**EUC_PS_243**	Greenwood	Greenwood	V	North America	Dry	ASR
**EUC_PS_244**	Kayanne	Kayanne	V	Denmark	Dry	ASR
**EUC_PS_245**	Solido	Solido	V	Netherlands	Marrowfat	ASR
**EUC_PS_246**	yellowstone	yellowstone	V	North America	Dry	ASR
**EUC_PS_247**	KARAT	KARAT	V	Serbia	Dry	IFVCNS
**EUC_PS_248**	PARTNER	PARTNER	V	Serbia	Dry	IFVCNS
**EUC_PS_249**	DUKAT	DUKAT	V	Serbia	Dry	IFVCNS
**EUC_PS_250**	JUNIOR	JUNIOR	V	Serbia	Forage	IFVCNS
**EUC_PS_252**	A10Z**	A10Z**	BL	Serbia	Dry	IFVCNS
**EUC_PS_253**	KOSMAJ	KOSMAJ	V	Serbia	Forage	IFVCNS
**EUC_PS_254**	Mak 116	Mak116	V	Serbia	Dry	IFVCNS
**EUC_PS_255**	UKR 015A	UKR015A	V	Serbia	Dry	IFVCNS
**EUC_PS_256**	UKR 133S	UKR133S	V	Serbia	Dry	IFVCNS

**EUC_PS_257**	UKR 223A	UKR223A	V	Serbia	Dry	IFVCNS
**EUC_PS_258**	UKR 138S	UKR138S	V	Serbia	Dry	IFVCNS
**EUC_PS_259**	UKR 101I	UKR101I	V	Serbia	Dry	IFVCNS
**EUC_PS_260**	UKR 134G	UKR134G	V	Serbia	Dry	IFVCNS

* BL—breeding Line; V—variety; SW—semi-wild; W—wild; L—landrace; GS—genetic stock.

** ASR—Agro Seed Research, Kessenich; IBERS—Institute of Biological, Environmental and Rural Sciences, Aberystwyth; NorthGen—NorthGen Genetic Resource Center, Alnarp; IFVCNS—Institute of Field and Vegetable Crops, Novi Sad
